# Improved response of human gingival fibroblasts to titanium coated with micro-/nano-structured tantalum

**DOI:** 10.1186/s40729-021-00316-z

**Published:** 2021-05-03

**Authors:** Chu-nan Zhang, Lin-yi Zhou, Shu-jiao Qian, Ying-xin Gu, Jun-yu Shi, Hong-chang Lai

**Affiliations:** Department of Dental Implantology, Shanghai Ninth People’s Hospital, College of Stomatology, Shanghai Jiaotong University School of Medicine, Shanghai, 200011 China

**Keywords:** Tantalum-coating, Dental implants, Soft tissue integration, Integrin β1, Focal adhesion kinase

## Abstract

**Objectives:**

This study aims to evaluate the ability of tantalum-coated titanium to improve human gingival fibroblasts’ adhesion, viability, proliferation, migration performance, and the potential molecular mechanisms.

**Materials and methods:**

Titanium plates were divided into two groups: (1) no coating (Ti, control), (2) Tantalum-coated titanium (Ta-coated Ti). All samples were characterized by scanning electronic microscopy, surface roughness, and hydrophilicity. Fibroblasts’ performance were analyzed by attached cell number at 1 h, 4 h, and 24 h, morphology at 1 h and 4 h, viability at 1 day, 3 days, 5 days, and 7 days, recovery after wounding at 6 h, 12 h, and 24 h. RT-PCR, western blot were applied to detect attachment-related genes’ expression and protein synthesis at 4 h and 24 h. Student’s *t* test was used for statistical analysis.

**Results:**

Tantalum-coated titanium demonstrates a layer of homogeneously distributed nano-grains with mean diameter of 25.98 (± 14.75) nm. It was found that after tantalum deposition, human gingival fibroblasts (HGFs) adhesion, viability, proliferation, and migration were promoted in comparison to the control group. An upregulated level of Integrin β1 and FAK signaling was also detected, which might be the underlying mechanism.

**Conclusion:**

In the present study, adhesion, viability, proliferation, migration of human gingival fibroblasts are promoted on tantalum-coated titanium, upregulated integrin β1 and FAK might contribute to its superior performance, indicating tantalum coating can be applied in transmucosal part of dental implant.

**Clinical significance:**

Tantalum deposition on titanium surfaces can promote human gingival fibroblast adhesion, accordingly forming a well-organized soft tissue sealing and may contribute to a successful osseointegration.

## Introduction

**Fig. 1:**
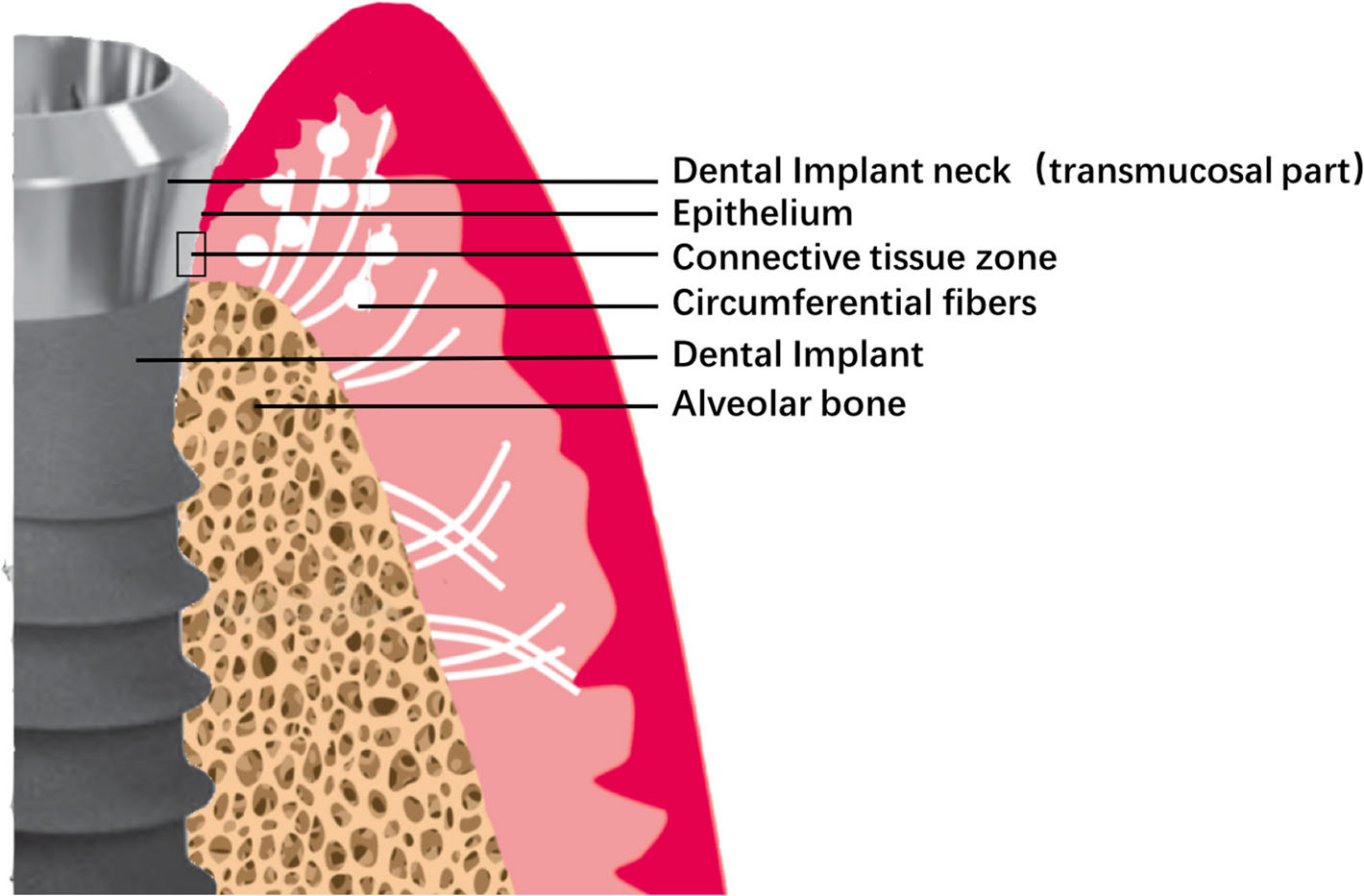
Schematic illustration of soft tissue implant integration. The gingival fibers in a parallel orientation on implant surface

After dental implant surgery, the following soft tissue healing constitutes a collar like seal, an effective physical and physiological barrier between oral environment and peri-implant bone. Soft tissue integration consists of epithelium and connective tissue [1]. However, due to lack of periodontal ligament, dental implant is more prone to have penetration of bacterial, as well as epithelial downgrowth [2] and invasion of all sorts of infections and toxins. Unsuccessful attachment of connective tissue to the implant can lead to bone loss and implant failure. Therefore, connective tissue seal around dental implant is essential for preventing peri-implantitis [3, 4] and maintaining successful osseointegration [5]. It has been proven that in a 300–600-μm-wide zone that connective tissue attached to traditional titanium implant surface, collagen (82.36%) was prone to appear in peripheral areas (40–200 μm) while cells (fibroblasts = 32.32%) appeared to be more closed to the implant surface in a narrow region (0–40 μm) [6] (Fig. 1). Thus, conclusions were drawn that gingival fibroblasts attached to implant surface plays an important role in establishing and maintaining adequate seal against external environment [7, 8].

Bacterial infection is the major factor that influences soft tissue integration [9]. Toxins from microflora like lipopolysaccharide would interfere with cell proliferation and differentiation and even induce cell pyroptosis. Various surface modification treatments have been applied to reduce biofilm formation on dental implant surface. For example, deposition of silver ion and titanium (zirconium) nitride, coating of silica, chitosan-lauric acid, and peptides [10–15]. However, most nanoparticle coatings with antibacterial effect are toxic and can cause foreign body response [16, 17]. The possible reason could be releasing of ions after interaction between titanium-based materials and human body fluid, which would cause poisoning, and allergy [18, 19].

With superior biocompatibility, physicochemical stability and corrosion resistance [20–23], tantalum coating exhibits effective antimicrobial activity against soft tissue infections [24, 25]. It can enhance phagocytosis of bacteria by polymorphonuclear neutrophils (PMNs, neutrophils), reduce lysis of neutrophils, and enhance macrophages releasing proinflammatory cytokine [24]. It was found that titanium have no multi-nucleated macrophages in the tissue surrounding the metallic implant, while tantalum implants displayed an occasional peri-implant macrophage [26]. Besides, studies have shown that tantalum implants placed in dorsal subcutaneous tissue demonstrate substantial attachment strength at 4 weeks, indicating tantalum has superior character for soft tissue attachment [27]. However, the effectiveness of tantalum coating on the connective-tissue integration of titanium implants remains unclear, let alone its relative mechanisms.

In addition, tantalum coating with its self-passivating surface oxide layer also possess super performance of anticorrosion and good wear resistance. However, unlike titanium, high modulus of elasticity and low frictional characteristics make it unsuitable for pure tantalum implant manufacturing. Here we applied magnetron sputtering technique to fabricate tantalum coating on titanium plates. It is an easy-controlling coating fabrication technique. Coatings fabricated by magnetron sputtering have high density and uniformity, as well as strong adhesion, low processing temperature [28]. It is a cost-effective technique.

Therefore, the aim of present study was to explore gingival fibroblasts adhesion, viability, proliferation, migration ability on tantalum coating on titanium in comparison with titanium, and its relative mechanism. Molecular level of specific cell adhesion-related genes expressions was detected. We aimed to figure out the clinical application potential of tantalum coatings in connective tissue integration aspects. The null hypotheses in this study were that tantalum coating cannot improve gingival fibroblasts adhesion, viability, proliferation, and migration on titanium plates.

## Materials and methods

### Preparation and characterization of materials

Commercial pure titanium plates (diameter 15 mm, thickness 1 mm) of grade IV were provided by Trausim Medical Instrument Co., Ltd, (Changzhou City, Jiangsu Province, People’s Republic of China) Tantalum was implanted onto titanium disk by magnetron-sputtering technique. Titanium plates were sputter-cleaned for 5 min at a bias of 800 V, duty factor of 30% and working pressure of 0.02 Pa. And then sputtered with Ti for 10 min at a bias of 250 V, duty factor of 30% and working pressure of 0.02 Pa. Then, tantalum deposition was done for 40 min by sputtering at a substrate bias of 150 V, duty factor of 80%, working pressure of 0.02 Pa.

Field-emission scanning electron microscopy (FE-SEM, JSM-6700F, JEOL Ltd., Japan) was used to observe the surface morphologies of two samples. Profilometer (Mahr Perthometer M1, Germany) was used to assess the surface roughness, and goniometer (OCA 15EC, Germany) was used to detect hydrophilicity of two surfaces, respectively.

### Cell culture of human gingival fibroblasts

Primary human gingival fibroblasts (HGFs) were obtained from healthy patients’ gingival biopsies. All experimental protocols were approved by the Independent Ethics Committee of *** School of Medicine. The collected tissues were rinsed with sterile phosphate buffered saline (PBS) supplemented with 2% antibiotics immediately after separation from gingiva and incubated in 2.0U/mL dispase II (Roche, Basel, Switzerland) for 40 min at 37 °C in order to separate the epithelium layer from the underlying connective tissue. Then tissues were cut into 1.0 mm^3^ small pieces with sterile scissors. After being dampened with Dulbecco’s modified Eagle medium (DMEM; GIBCO Laboratories, Grand Island, NY), tissue fragments were evenly scattered on the dish. Then, the dish was inverted in an incubator for 4 h. Finally, the dish was flipped upright and filled with 10 mL DMEM containing 10% fetal bovine serum (FBS, GIBCO), 1% penicillin/streptomycin and 2 mM glutamine at 37°C in a 5% CO2 humidified atmosphere. It took about 2 weeks for the primary cells to reach confluence, and then, the cells were washed with PBS and passaged with 0.25% trypsin/ethylenediaminetetraacetic acid (trypsin/EDTA). Cells from passages 2–6 were used for the experiments.

### Cell adhesion ability assay

Cell numbers in the initial seeding period (1, 4, and 24 h) were measured to represent the adhesive cells on different samples. HGFs at a density of 5 × 10^4^ cells per well were incubated on three samples at each time point for each group in 24-well plates. And the experiment was performed three times (*n* = 27 per group). At each time point, cells remained on the plates were fixed with 4% paraformaldehyde at 4 °C. Cell nuclei were stained with DAPI for 5 min at room temperature and then observed under a confocal laser-scanning microscope. Each sample was selected to obtain a relatively uniform distribution of three different horizons for images. To count the number of cells attached.

### Cell morphological observation by scanning electron microscopy (SEM)

Field-emission scanning electron microscopy (FE-SEM, JSM-6700F, JEOL Ltd, Japan) was applied to observe the cell adhesion morphology. Briefly, the HGFs were seeded on three samples at each time point for each group in a 24-well plate at a density of 1 × 10^4^/well and repeated three times (*n* = 18 per group). After incubating for 1, 4 h, all samples were fixed with 2.5% glutaraldehyde overnight at 4°C. Ethanol with a series of concentration gradient of 30, 50, 75, 90, 95, and 100 v/v% was used sequentially to dehydrate the samples. The samples were finally dried in a fume cupboard. All samples were sputter coated with gold for observation.

### Cell viability and proliferation

Viability of HGFs was evaluated using a Live/Dead viability kit (Biovision, USA). The HGFs were seeded on three samples per group in 24-well plates at a density of 1 × 10^5^ cells per well and the experiment was performed three times (*n* = 9 per group). After 24 h, the samples were rinsed with PBS and then incubated in the Live/Dead solution (2 μM Calcein-AM and 4 μM PI) for 15 min at 37 °C

The Counting Kit-8 (CCK-8, Dojindo Laboratories Inc., Kumamoto, Japan) assay was used to evaluate the proliferation activity of HGFs on different samples. Initially, 5 × 10^4^ cells/mL was seeded onto three samples per group in 24-well plates and cultured for 1, 3, 5, and 7 days, respectively. And the experiment was carried out three times (*n* = 36 per group). At each time point, three samples per group were washed twice with PBS and then incubated in 600 μL of DMEM with a supplement of 60 μL of CCK-8 solution for an hour. The collected solution was carefully transferred to a 96-well plate with 200 μL per well. The absorbance was read at the wavelength of 450 nm according to the manufacturer’s instructions by the microplate spectrophotometer (Bio Tek, Highland Park, Winooski, USA). The results were shown as units of optical density (OD) absorbance value.

### Wound healing assay

Wound healing assay was conducted to study the cell migration ability on both samples. Briefly, 1 × 10^5^ cells per milliliter were grown on three samples per group and allowed to reach 100% confluence. Experiment was performed three times (*n* = 36 per group). Before being wounded, the samples were incubated overnight in a medium containing 2% FBS, and then, the cell monolayers were carefully wounded with a plastic pipette. Images were taken at 0 h, 6 h, 12 h, and 24 h after the cells were wounded. At each time point, samples from both groups were transferred to a new plate and fixed with 4% paraformaldehyde for 30 min. Cells were permeated by 0.1% Triton X-100 for 10 min and incubated with Rhodamine-phalloidin (Enzo Life Sciences, Exeter, UK) for 30 min at room temperature after being washed three times with PBS. Finally, samples were incubated in DAPI (Solarbio, China) for 5 min. Images of cells on all samples were taken using confocal laser-scanning microscope (Leica, Hamburg, Germany). The regions between the cell layer borders were measured using NIH ImageJ software. Wound healing percentage was calculated based on initial wound region.

### Immunofluorescence of adhesion-related proteins

HGFs were seeded at a density of 1 × 10^4^ cells per well on three samples per group and incubated for 4 and 24 h in 24-well plates. Experiment was repeated three times (*n* = 18 per group per protein). After washing with PBS twice, the remained cells were fixed in 4% paraformaldehyde for 30 min at 4 °C and then treated with 0.1% Triton X-100 for 10 min. Then, after being blocked by bovine serum albumin, BSA (1 wt% in PBS, Sigma Aldrich, MO, USA) for 1 h, the samples were incubated with specific primary antibody targeting Integrin β1 and Vinculin (Abcam, Cambridge) at room temperature for 1 h, respectively. After twice PBS washes, all samples were incubated in DyLight 488-conjugated anti-mouse IgG antibody (Invitrogen) for another hour at room temperature in the dark. Then, a rhodamine-phalloidin antibody was applied to the samples for 1 h in dark. The nuclei were stained with DAPI for another 5 min after twice washing with PBS, and all specimens were observed using Zeiss 710 confocal laser-scanning microscope.

### RNA isolation and gene expression by quantitative real-time PCR analysis

Real-time PCR assay was performed to investigate relative gene expression level of HGFs seeded on CFRPEEK plates. Cells were seeded on three samples (15 mm × 15 mm × 1 mm) per group in 6-well plates with 2 × 10^5^ cells per milliliter and incubated for 4 and 24 h. The experiment was carried out three times (*n* = 18 per group). The total RNA was extracted using TRIzol reagent (Invitrogen, Carlsbad, USA), and cDNA was generated using PrimeScript 1st Strand cDNA Synthesis kit (TaKaRa, Japan) according to the manufacturer’s instructions. The gene expression of Vinculin (VCL), Integrin β1 (ITGB1), Integrin α2 (ITGA2), Integrin α5 (ΙΤGΑ5), collagen type 1 (Col-1A1), and Fibronectin (FN) were detected by the Bio-Rad Quantitative Real time PCR system (qRT-PCR; Bio-Rad, MyiQ, USA). Specific gene primers were synthesized commercially (Shenggong Co., Ltd. Shanghai, China), and the genes, primer sequences, and amplicon sizes were listed in Table 1. All mRNA values were normalized against glyceraldehyde 3-phosphate dehydrogenase (GAPDH) expression.

**Table 1:** Primer pairs used in real-time PCR analysis

Gene	Primers (F = forward, R = reverse)	Amplicon
ITGB1	F:TGGAGGAAATGGTGTTTGC	107 bp
	R:CGTTGCTGGCTTCACAAGTA	
ITGA2	F:GCACCACATTAGCATACAGA	138 bp
	R:GGCATCATACAGGAGAGGAA	
ITGA5	F:GGCTGTGTATGGGGAGAAGA	200 bp
	R:TCACCGCGAAGTAGTCACAG	
VCL	F:CGAATCCCAACCATAAGCAC	158 bp
	R:CGCACAGTCTCCTTCACAGA	
FN1	F:GACCGAAATCACAGCCAGTAG	101 bp
	R:CATCTCCCTCCTCACTCAGC	
COL-1A1	F:AAGACATCCCACCAATCACC	120 bp
	R:CGTCATCGCACAACACCTT	
GAPDH	F:TGTGTCCGTCGTGGATCTGA	150 bp
	R:TTGCTGTTGAAGTCGCAGGAG	

### Western blot

For western blot, after seeding at a density of 2 × 10^5^ cells/mL on 6 samples per group in 6 cm cell culture dishes at 4 and 24 h and repeated three times (*n* = 36 per group), HGFs were collected and lysed with a protein extraction regent containing protease inhibitor, phosphatase inhibitor and phenylmethanesulfonyl fluoride (PMSF). The protein concentration was measured using a Bio-Rad protein assay kit. Equal amounts of protein (40 μg/lane) were separated on SDS-polyacrylamide gel electrophoresis and electrotransferred to a polyvinylidene difluoride (PVDF) membrane (Millipore). Membranes were incubated mouse antibody FAK, phospho-FAK (Tyr397), and β-actin (CST, 1:1000 dilution) overnight at 4 °C. Afterwards, the primary antibodies were detected by horseradish peroxidase-conjugated goat anti-rabbit and goat anti-mouse in PBS for 60 min. Bound secondary antibodies were visualized by enhanced chemiluminescence (ECL). The protein bands were quantitated by the ImageJ.

### Fibronectin adsorption on surfaces

Here, we use ELISA to measure the amount of fibronectin adsorbed on the titanium and Ta-coated Ti surfaces. Three samples per group were placed in 24-well plates and 1 mL DMEM supplemented with 10% FBS was added for 1 and 4 h. The experiment was carried out three times (*n* = 18 per group). After rinsing with PBS, all samples were incubated in 1% BSA for 1 h, then treated with rabbit anti-fibronectin primary antibody (Proteintech, dilution, 1:50) for 1 h at room temperature. After rinsing 3 times with 0.1% Tween 20 for 15 min, samples were incubated for 45 min with anti-rabbit secondary antibody conjugated with horseradish peroxidase (Jackson ImmunoResearch Laboratories, Inc., Baltimore, USA). Finally, the amount of fibronectin adsorbed to the surface was measured with the TMB (QuantiCyto®, Shenzhen, China). Light absorbance was measured at 450 nm. Both samples were performed in triplicate, and the results were shown as units of optical density (OD) absorbance value.

### Statistical analysis

All experiments were conducted in triplicate. The data were shown as mean ± standard deviation. Student’s *t* test was applied to analyze the statistical difference between two groups. The significant difference was set at a level of *p* < 0.05. All of the statistical analyses were determined with the GraphPad Prism 8 statistical software package.

## Results

### Surface characterization

**Fig. 2:**
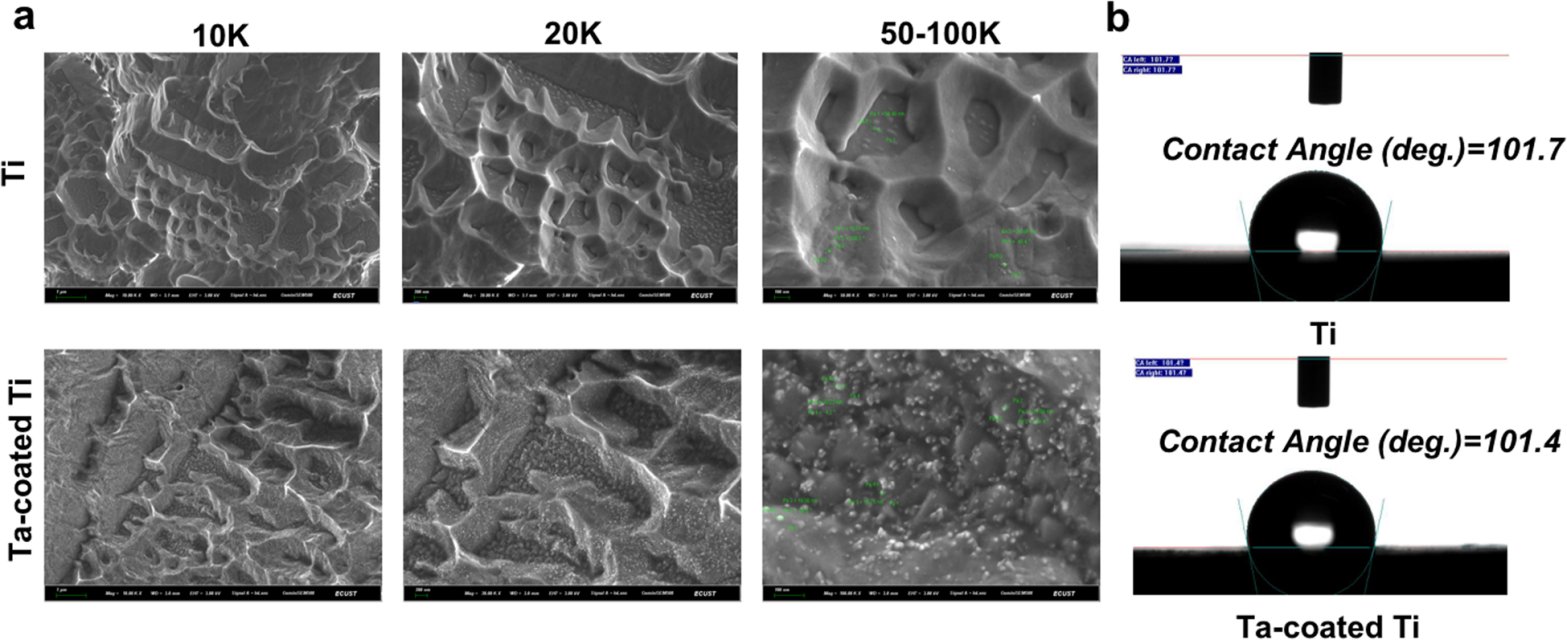
**a** SEM morphologies of Ti an Ta-coated Ti plates under different magnifications. **b** Surface wettability. No obvious difference for contact angles (*p* > 0.05)

Surface characterization of the two samples at 0 h was shown in Fig. 2a. Macro-pits and micro-pits were seen on the surfaces of the two samples. After tantalum deposition, nano-grains ranged from 16 to 30 nm can be observed distributing homogeneously and forming a cover. Surface roughness were 1.028 ± 0.051 μm for titanium plates and 0.8183 ± 0.057 μm for Ta-coated Ti plates. Average contact angle on Ti plates was 101.7 ± 0.125°, while Ta-coated ones were 101.4 ± 0.163°, which is not statistically different.

### Early cell adhesion

**Fig. 3:**
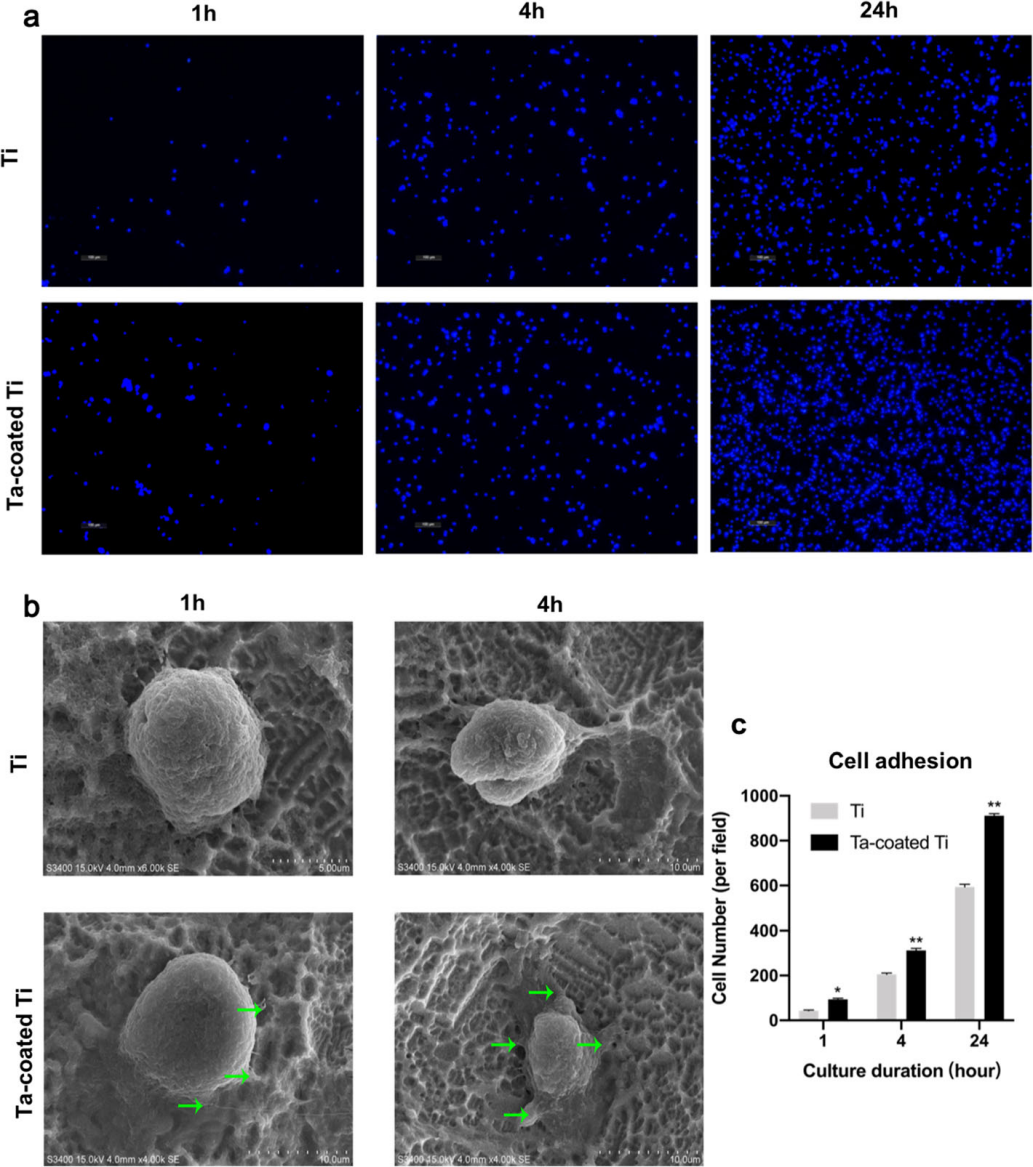
**a** Cell adhesion assy. Cell nuclei stained with DAPI on both samples were imaged by confocal laser scanning microscopy at 1, 4, and 24 h after seeding (× 100). **b** SEM morphology of HGFs adhered on tantalum-coated titanium and titanium plates at 1 h and 4 h, noting that cells on Ta-coated Ti plates at 4 h with more extensively spread cytoplasm than those on Ti plates, the green arrow represented the filopodium-like projections of spreading gingival fibroblast cells. **c** Statistical results of adhesive cell numbers. *p < 0.001, **p < 0.0001 when compared with Ti group

As shown in Fig. 3a, the numbers of HGFs on Ta-coated Ti plates overtake those attached to the Ti groups after incubation for 1, 4, 24 h. The statistical analysis of cell counting results also indicate more adherent cells on Ta-coated Ti with 93.67 ± 4.11 at 1 h, 311.67 ± 7.85 at 4 h, and 911.33 ± 7.41 at 24 h than Ti with 42.67 ± 2.87 at 1 h, 205.33 ± 4.64 at 4 h, and 593.33 ± 10.08 at 24 h, especially at 24 h (*p* < 0.05) (Fig. 3c).

### SEM analysis of cell morphology

SEM morphologies of HGFs cultured on two samples for 1, 4 h are shown in Fig. 3b. After 1 h-seeding, cells on Ti plates demonstrate spherical appearance while cells on Ta-coated Ti plates have more filopodium-like projections indicated with green arrows. After 4 h-seeding, cells on Ti plates begin showing filopodium-like projections while cells on Ta-coated Ti plates have spreaded cytoplasm bound tightly to the surface. These results demonstrate tantalum coating promotes HGFs adherence.

### Cell viability and proliferation ability

**Fig. 4:**
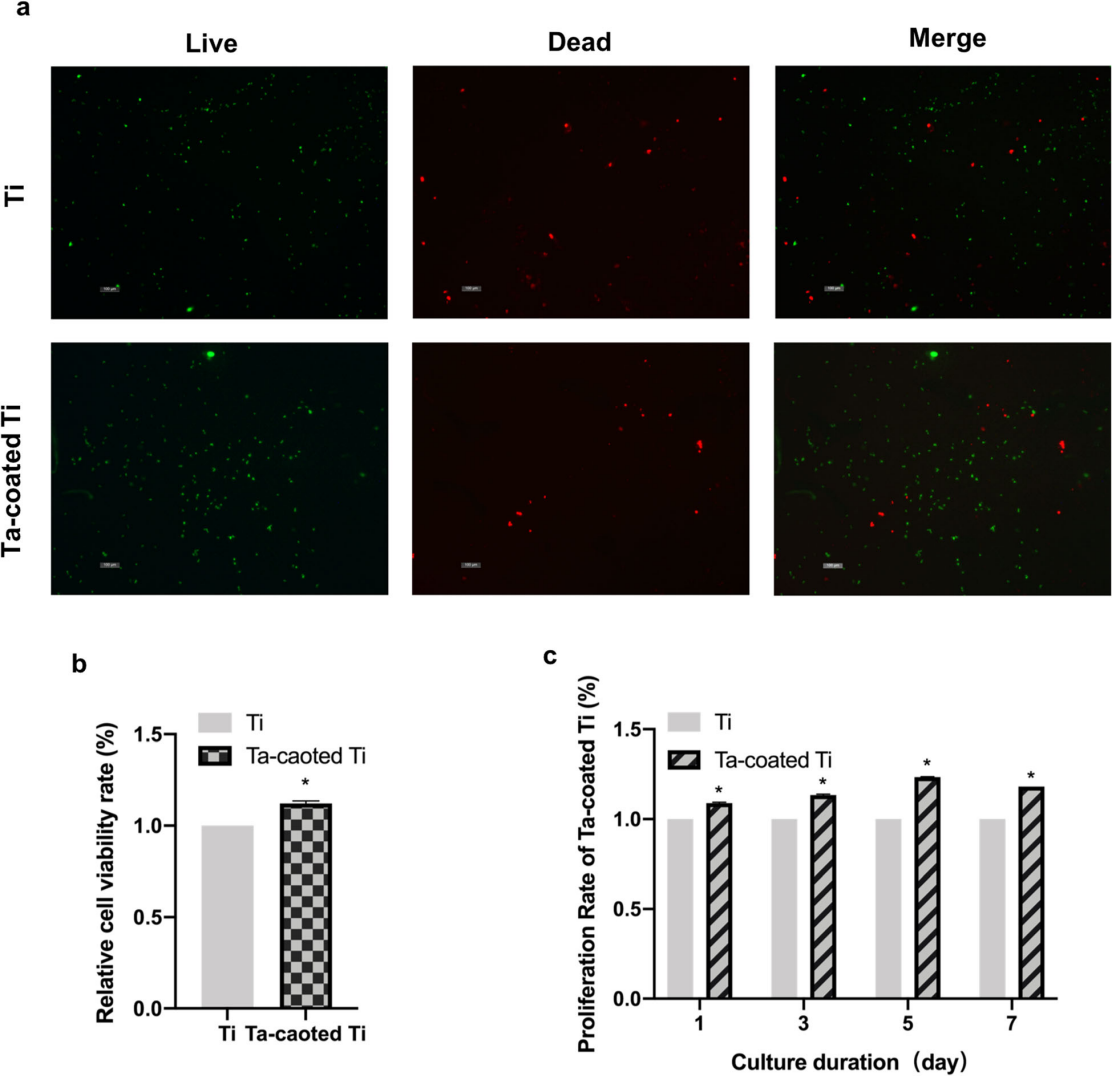
Relative cell viability and proliferation assay. **a** HGFs cultured on both samples for 24 h were stained with Calcein AM (green) and PI (red), indicating live and dead cells (× 100). **b** Relative HGF viability rate was detected at 24h based on live/dead cell staining. *p < 0.001 when compared with Ti group. **c** Relative HGFs proliferation rate on both samples was detected by CCK-8 at 1, 3, 5, and 7 days. *p < 0.0001 when compared with Ti group

As shown in Fig. 4a, the proportion of dead cells after seeding for 24 h was low (red fluorescence), suggesting most of the cells maintained their viability on the surface and both samples have good biocompatibility to HGFs. Relative HGF viability rate is exhibited in Fig. 4b with 1.12 ± 0.01 for Ta-coated Ti. Relative proliferation rate of HGFs cultured on both Ti and Ta-coated Ti is exhibited in Fig. 4c. Proliferation ability of HGFs on Ta-coated Ti plates with 1.09 ± 0.004 at day 1, 1.13 ± 0.004 at day 2, 1.23 ± 0.002 at day 5, 1.18 ± 0.0001 at day 7 is always superior to those on Ti plates.

### Wound healing assay

**Fig. 5:**
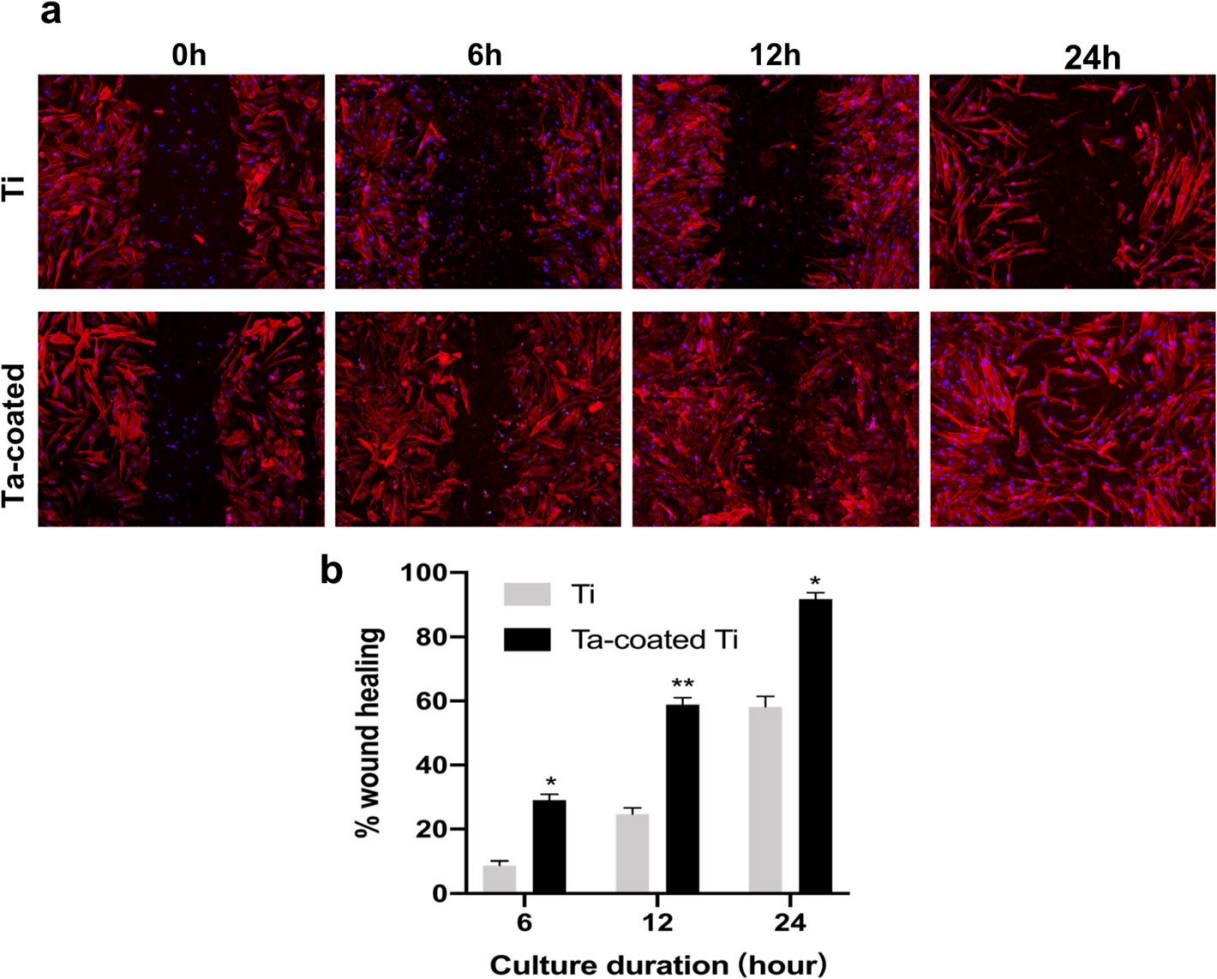
Wound healing assay of HGFs on both samples at 0, 6, 12, and 24 h. **a** Nuclei (blue) and actin filaments (red) were visualized by DAPI and rhodamine-phalloidin (× 100). **b** The quantification of wound heal percentages.*p < 0.001, **p < 0.0001 when compared with the Ti group

As shown in Fig. 5a, Ta-coated Ti plates promote cell migration better than Ti plates. For titanium plates, only 8.76 ± 1.12% of the wound was closed after 6 h while 29.03 ± 1.47% of the wound was closed for Ta-coated Ti plates, and 24.73 ± 1.58% for Ti plates, 58.91 ± 1.72% for Ta-coated Ti at 12 h; 58.17 ± 2.67% for Ti plates, 91.73 ± 1.64% for Ta-coated Ti at 24 h. After 24 h, HGFs on Ta-coated Ti demonstrated a formed cellular monolayer.

### Immunofluorescence of adhesion-related proteins

**Fig. 6:**
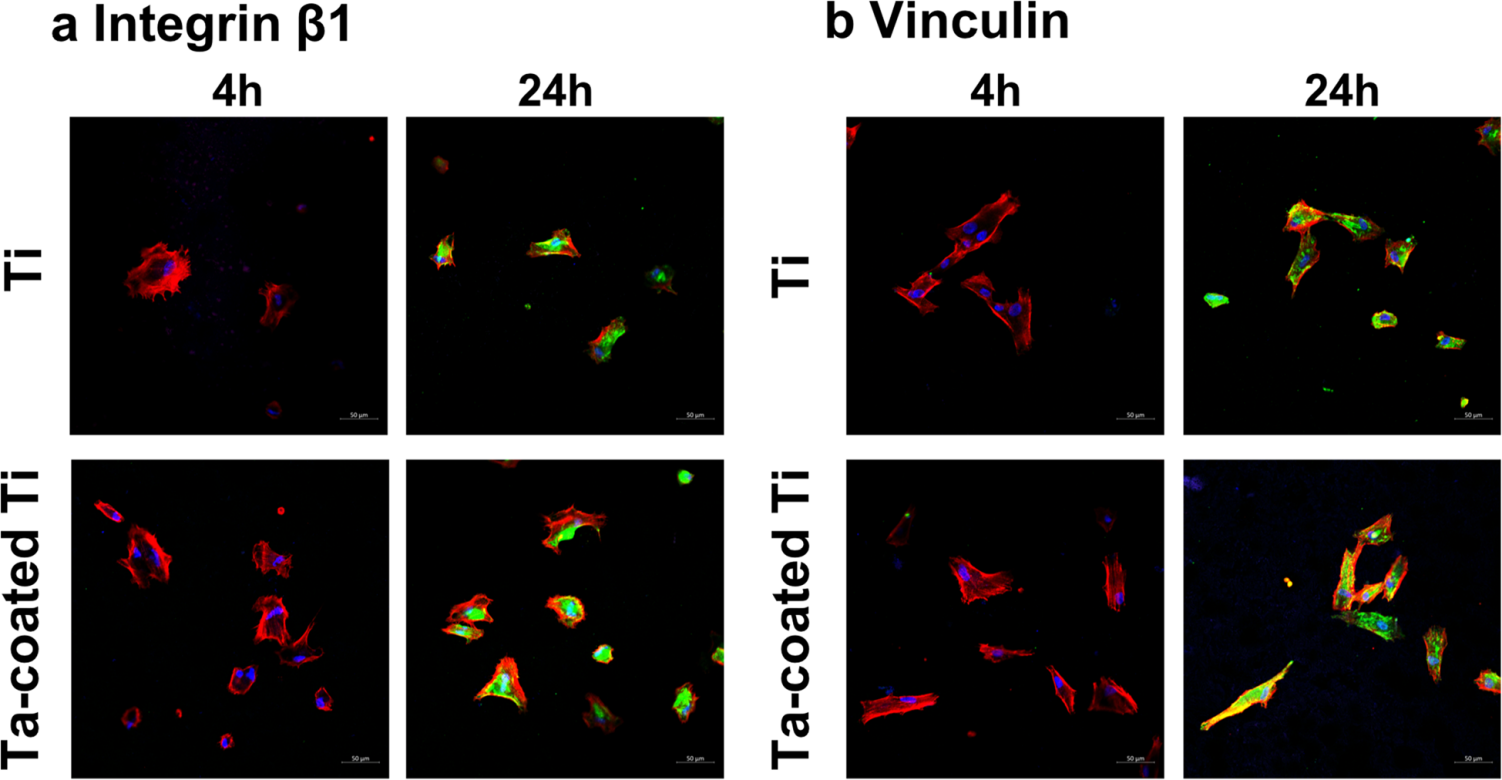
Immunofluorescence expression of adhesion-related proteins detected by confocal laser scanning microscopy. **a** Expression of integrin β1 (green) of HGFs from titanium plates and tantalum coated titanium plates at 4 h and 24 h after seeding. **b** Expression of Vinculin (green) of HGFs from titanium plates and tantalum coated titanium plates at 4 h and 24 h after seeding

Immunofluorescence of adhesion-related proteins vinculin and integrin β1 was performed to further explore adhesion ability of HGFs on both samples. As shown in Fig. 6a, HGFs adhering to Ta-coated Ti plates express more vinculin at 24 h. While integrin β1 of HGFs on Ta-coated Ti plates is also increasingly distributed compared with those on Ti plates.

### Adhesion-related genes analysis

**Fig. 7:**
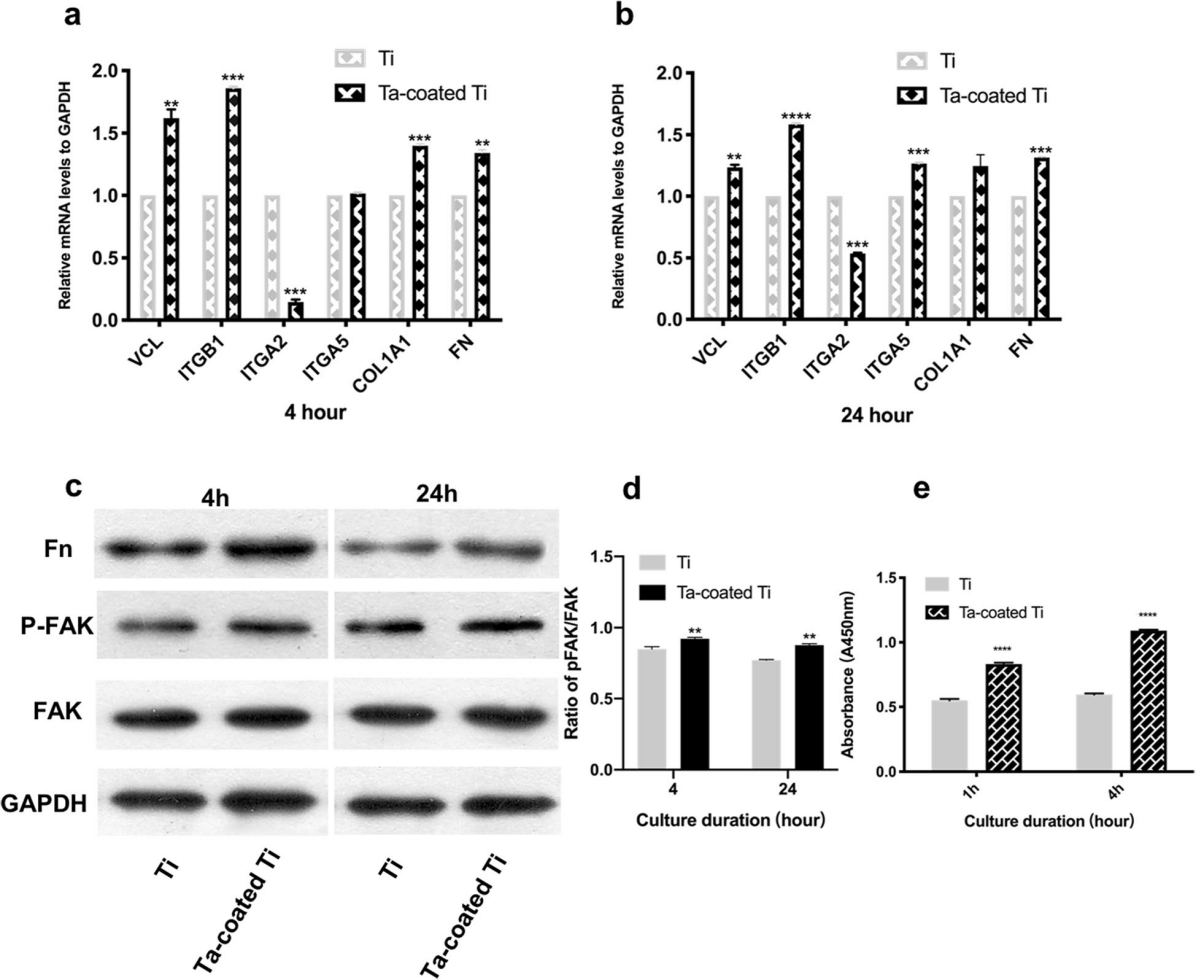
**a**, **b** Real-time PCR detection of adhesion-related gene expression of HGFs on both samples at 4 and 24 h with statistical significance by **p < 0.01, ***p < 0.001, ****p < 0.0001. **c** Western blot for signaling protein (FAK and pFAK) and Fn. **d** The expression ratio of p-FAK/FAK quantified by densitometry (*n* = 3).**p < 0.01. **e** Increased fibronectin adsorption on Ta-coated Ti surface compared to Ti group (****p < 0.0001)

Figure 7a, b exhibits the HGFs mRNA expression of vinculin, integrin β1, integrin α2, integrin α5, collagen type 1 and fibronectin on both samples at 4 h and 24 h. HGFs from Ta-coated Ti plates showed higher vinculin and Integrin β1 gene expression, compared with HGFs from titanium plates, which is in accordance with the results of immunofluorescence. Gene expression of collagen type 1 is also higher for HGFs from Ta-coated Ti plates, and showed a significant difference at 4 h.

### Western blot

Western blot was applied to investigate FAK expression and phosphorylation for HGFs on both surfaces. As shown in Fig. 7c, higher levels of phosphor-FAK proteins with gray value of 0.92 ± 0.01 at 4 h and 0.88 ± 0.01 at 24 h are registered for HGFs on Ta-coated Ti plates at both time points. Meanwhile, fibronectin expression is also increased on Ta-coated Ti plates.

### Protein adsorption

Figure 7e shows fibronectin adsorption of two samples in 1 h and 4 h. Ta-Ti plates with absorbance of 0.83 ± 0.01 at 1 h and 1.09 ± 0.01 at 4 h demonstrate relatively higher adsorption than Ti plates with 0.55 ± 0.01 at 1 h and 0.60 ± 0.01 at 4 h, and there is a statistical significance at both time points, implying tantalum coating promotes adsorption of fibronectin (*p* < 0.05).

## Discussion

The objective of this study was to investigate the ability of tantalum coating on titanium to promote HGFs adhesion, viability, proliferation, and migration. And we found out that tantalum coating can promote HGFs adhesion, proliferation, and migration without reducing viability, which can be ascribed to increased expression of integrin β1 and activation of FAK signaling.

Surface roughness and hydrophobicity, the predominant characteristics of implant surface, are considered as key-properties of dental implant surfaces for both tissue integration [29, 30], and biofilm formation [31]. Here, we applied magnetron sputtering technique to acquire tantalum (Ta) films deposited on titanium surfaces, which possess high bonding strengths between deposited layers and substrates. Studies have shown that fibroblasts prefer smooth surfaces rather than roughened ones [32]. In this study, after tantalum deposition on titanium disks, surfaces with tantalum coating become relatively smoother, and better performance of HGFs adhesion, proliferation, and migration were detected.

Conclusions have been extracted from investigations that hydrophilicity rather than topography is more likely to influence soft-tissue integration, and hydrophilic surfaces can promote more intimate connective tissue attachment [33, 34], support the wound healing and thus accelerating osseointegration [28]. Though it was suggested that material surfaces with contact angles between 30° and 60° favor serum protein adsorption, protein exchange with cell adhesive serum proteins [35]. Ηigh surface energy may also promote cell adhesion and proliferation [36]. Ηοwever, it was also suggested that surfaces with higher surface energy are more preferred for bacterial adherence. Contact angles analysis here have shown that it is not statistically different between tantalum-coated titanium samples and titanium samples, indicating tantalum coating does not change surface hydrophilicity, superior connective tissue integration mainly ascribes to chemical trait of tantalum. Besides, investigation showed that implant decorated Ta2O5 films hardly cause cytotoxity, allergy, and chronic inflammation [37]. Its superior anticorrosion property can even annul abnormal apoptosis caused by released ions.

It is asserted that soft tissue sealing forms prior to osteointegration. Cell adhesion determines the speed and condition of later phase of proliferation and migration [38]. In present study, tantalum-coated titanium showed faster and denser initial HGFs adhesion than pure titanium indicates its superior biocompatibility. Studies have shown that integrin as well as fibronectin assist cell adhesion [9]. Tantalum coatings obviously increased these genes expression. Wound healing assay provided evidence for tight adhesion and quick reliable soft tissue repair around the implant surface.

HGFs are the major constitutes of connective tissue around inserted implant. Nevertheless, in contrast to connective tissue attaching to natural teeth, where collagen fibers insert into root cementum in a perpendicular direction, fibers around implant are parallel to the surface [1]. They sustain tissue integrity by regulating collagen and proteoglycan metabolism [39]. Study shows that once tissue cells integrate the implant surface, they begin yielding protection protein and collagen against infection. Tissue integration to implant surface starts with a cascade of protein adsorption [40]. Collagen stem from ECM components is vital for HGFs adhesion, migration on material surfaces. The adsorbed proteins provide recognition sites for cell attachment [41]. It also directs tissue development and provides tensile strength [42]. Fibrinogen, vitronectin, and fibronectin play important roles in adhesive serum proteins. Fibronectin, as an extracellular matrix protein, provide structures for cell attachment, migration, and differentiation through transmembrane receptors integrins [43, 44]. Optimal activation of integrin will then enhance cell adhesion and proliferation on the surface. Integrins are also detected here. However, neither integrin α2 nor integrin α5 were detected increased. This may suggest that improvement of HGF adhesion to tantalum-coated titanium was mainly related with integrin β1 and Fn. The formed matrix through interaction between Fn and integrins served binding sites for regulatory factors. As a collagen receptor, integrin α2 functions during matrix remodeling and increases along with a fibrotic phenotype, which may clarify its low level. It has been suggested that higher deprotonation rate on the surface may attributed to fibronectin staying in a compact structure, which therefore would affect interaction with integrin which regulating cytoskeleton and mediating related signal pathway [45]. Intracellular focal adhesion protein vinculin connects integrins to actin filaments, as well as linking extracellular and cytoskeletal elements, is a major protein involved in cellular adhesion and can be applied to track the adhesion process. As the key role in stabilizing focal adhesion, it indicates the promoted ability of HGF adhering to tantalum-coated titanium surfaces.

The Ta-modified surfaces prominently improved the initial adhesion and spreading of HGFs, not only depend on the up-regulation of adhesion-related genes like integrin β1, but also related to the activation of integrin co-localized enzyme, focal adhesion kinase FAK [46]. Here, phosphorylation of FAK was detected at an early stage on Ta-coated surfaces, which increased the number of bound integrins and adhesion strength over time. The cell motility and attachment-related cell receptors can activate FAK signaling and then trigger downstream biochemical molecules. It is the possible signaling pathway that may explain the effect of tantalum coatings on cell adhesion. FAK is closely related to integrin-stimulated signaling events and is a downstream signaling molecule of integrin activation. As a cytoplasmic tyrosine kinase, it can be activated by autophosphorylation at tyrosine 397 upon integrin β1 activation. FAK can influence the dynamic regulation of integrin-associated cell adhesion by further activating downstream signaling molecules such as Src and PI3K. Moreover, actin cyto-skeleton can also be influenced; therefore, cell migration can be controlled in way of migrating cells’ focal-complex assembly/disassembly cycle. Besides, FAK phosphorylation can contribute to reconstruction of connective tissue during wound healing by activation of ERK and p38 and following promoting TGF-β1-induced α-SMA expression required for fibroblast differentiation.

For further studies, though in vivo study demonstrated that the surface characteristics, including roughness did not influence dimension and composition of peri-implant mucosal attachment [47], Nevins et al. reported that formation of perpendicularly attached connective tissue fibers to implant surface can be promoted with application of laser microgrooves [48–50]. Studies have shown finely grooved surfaces had better fibroblasts attachment performance than smooth ones [51–53]. It has been shown that fibroblasts prefer wormlike structures with average height of 3.2 nm rather than dot like nanostructures with average height of 6 nm, proved by earlier adhesion and promoted proliferation [54]. Further studies can be carried out about the fibroblast attachment ability of titanium coated with topography modified with wormlike structures tantalum.

## Conclusion

In summary, current study found out that nanoscale tantalum oxides coatings on titanium fabricated by DC magnetron sputtering technique showed better HGFs adhesion as well as promotion of cell proliferation and migration speed. Meanwhile increased expression of HGF integrin β1 and activation of FAK were detected, which may well explain the outstanding performance of HGFs on tantalum oxides-coated titanium surfaces, indicating enhancement of peri-implant soft-tissue integration for clinical application. However, further in vivo studies are needed to excavate its clinical application potential.

## Data Availability

The datasets used in the study are available from the corresponding author on reasonable request.

## Abbreviations

Ti: Titanium
Ta: Tantalum
PMNs: Polymorphonuclear neutrophils
FE-SEM: Field-emission scanning electron microscopy
HGFs: Primary human gingival fibroblasts
SEM: Scanning electron microscopy
VCL: Vinculin
ITGB1: Integrin β1
ITGA2: Integrin α2
ITGA5: Integrin α5
Col-1A1: Collagen type 1
FN: Fibronectin

## References

[1] Berglundh T, Lindhe J, Ericsson I, Marinello CP, Liljenberg B, Thomsen P (1991). The soft tissue barrier at implants and teeth. Clin Oral Implants Res.

[2] Chehroudi B, Gould TR, Brunette DM (1992). The role of connective tissue in inhibiting epithelial downgrowth on titanium-coated percutaneous implants. J Biomed Mater Res.

[3] Buser D, Weber HP, Donath K, Fiorellini JP, Paquette DW, Williams RC (1992). Soft tissue reactions to non-submerged unloaded titanium implants in beagle dogs. J Periodontol.

[4] Sculean A, Gruber R, Bosshardt DD (2014). Soft tissue wound healing around teeth and dental implants. J Clin Periodontol.

[5] Albrektsson T, Branemark PI, Hansson HA, Lindstrom J (1981). Osseointegrated titanium implants. Requirements for ensuring a long-lasting, direct bone-to-implant anchorage in man. Acta Orthop Scand.

[6] Abrahamsson I, Berglundh T, Wennstrom J, Lindhe J (1996). The peri-implant hard and soft tissues at different implant systems. A comparative study in the dog. Clin Oral Implants Res.

[7] Moon IS, Berglundh T, Abrahamsson I, Linder E, Lindhe J (1999). The barrier between the keratinized mucosa and the dental implant. An experimental study in the dog. J Clin Periodontol.

[8] Palaiologou AA, Yukna RA, Moses R, Lallier TE (2001). Gingival, dermal, and periodontal ligament fibroblasts express different extracellular matrix receptors. J Periodontol.

[9] Liu M, Zhou J, Yang Y, Zheng M, Yang J, Tan J (2015). Surface modification of zirconia with polydopamine to enhance fibroblast response and decrease bacterial activity in vitro: a potential technique for soft tissue engineering applications. Colloids Surf B Biointerfaces.

[10] Grossner-Schreiber B, Griepentrog M, Haustein I, Muller WD, Lange KP, Briedigkeit H, Gobel UB (2001). Plaque formation on surface modified dental implants. An in vitro study. Clin Oral Implants Res.

[11] Qin H, Cao H, Zhao Y, Zhu C, Cheng T, Wang Q, Peng X, Cheng M, Wang J, Jin G, Jiang Y, Zhang X, Liu X, Chu PK (2014). In vitro and in vivo anti-biofilm effects of silver nanoparticles immobilized on titanium. Biomaterials.

[12] Villard N, Seneviratne C, Tsoi JK, Heinonen M, Matinlinna J (2015). Candida albicans aspects of novel silane system-coated titanium and zirconia implant surfaces. Clin Oral Implants Res.

[13] Zhao L, Hu Y, Xu D, Cai K (2014). Surface functionalization of titanium substrates with chitosan-lauric acid conjugate to enhance osteoblasts functions and inhibit bacteria adhesion. Colloids Surf B Biointerfaces.

[14] Zhang F, Zhang Z, Zhu X, Kang ET, Neoh KG (2008). Silk-functionalized titanium surfaces for enhancing osteoblast functions and reducing bacterial adhesion. Biomaterials.

[15] Kazemzadeh-Narbat M, Lai BF, Ding C, Kizhakkedathu JN, Hancock RE, Wang R (2013). Multilayered coating on titanium for controlled release of antimicrobial peptides for the prevention of implant-associated infections. Biomaterials.

[16] Li YF, Chen C (2011). Fate and toxicity of metallic and metal-containing nanoparticles for biomedical applications. Small.

[17] Ma M, Liu WF, Hill PS, Bratlie KM, Siegwart DJ, Chin J, Park M, Guerreiro J, Anderson DG (2011). Development of cationic polymer coatings to regulate foreign-body responses. Adv Mater.

[18] Okazaki Y, Gotoh E (2005). Comparison of metal release from various metallic biomaterials in vitro. Biomaterials.

[19] Woodman JL, Jacobs JJ, Galante JO, Urban RM (1984). Metal ion release from titanium-based prosthetic segmental replacements of long bones in baboons: a long-term study. J Orthop Res.

[20] Alberius P (1983). Bone reactions to tantalum markers. A scanning electron microscopic study. Acta Anat (Basel).

[21] Black J (1994). Biological performance of tantalum. Clin Mater.

[22] Aronson AS, Jonsson N, Alberius P (1985). Tantalum markers in radiography. An assessment of tissue reactions. Skeletal Radiol.

[23] Robinson KA, Roubin GS, King SB (1996). Long-term intracoronary stent placement: arteriographic and histologic results after 7 years in a dog model. Cathet Cardiovasc Diagn.

[24] Yang C, Li J, Zhu C, Zhang Q, Yu J, Wang J, Wang Q, Tang J, Zhou H, Shen H (2019). Advanced antibacterial activity of biocompatible tantalum nanofilm via enhanced local innate immunity. Acta Biomater.

[25] Zhang XM, Li Y, Gu YX, Zhang CN, Lai HC, Shi JY (2019). Ta-coated titanium surface with superior bacteriostasis and osseointegration. Int J Nanomedicine.

[26] Johansson CB, Hansson HA, Albrektsson T (1990). Qualitative interfacial study between bone and tantalum, niobium or commercially pure titanium. Biomaterials.

[27] Hacking SA, Bobyn JD, Toh K, Tanzer M, Krygier JJ (2000). Fibrous tissue ingrowth and attachment to porous tantalum. J Biomed Mater Res.

[28] Rupp F, Liang L, Geis-Gerstorfer J, Scheideler L, Huttig F (2018). Surface characteristics of dental implants: a review. Dent Mater.

[29] Kloss FR, Steinmuller-Nethl D, Stigler RG, Ennemoser T, Rasse M, Hachl O (2011). In vivo investigation on connective tissue healing to polished surfaces with different surface wettability. Clin Oral Implants Res.

[30] Ma Q, Mei S, Ji K, Zhang Y, Chu PK (2011). Immobilization of Ag nanoparticles/FGF-2 on a modified titanium implant surface and improved human gingival fibroblasts behavior. J Biomed Mater Res A.

[31] Teughels W, Van Assche N, Sliepen I, Quirynen M (2006). Effect of material characteristics and/or surface topography on biofilm development. Clin Oral Implants Res.

[32] Wieland M, Chehroudi B, Textor M, Brunette DM (2002). Use of Ti-coated replicas to investigate the effects on fibroblast shape of surfaces with varying roughness and constant chemical composition. J Biomed Mater Res.

[33] Schwarz F, Herten M, Sager M, Wieland M, Dard M, Becker J (2007). Histological and immunohistochemical analysis of initial and early subepithelial connective tissue attachment at chemically modified and conventional SLA titanium implants. A pilot study in dogs. Clin Oral Investig.

[34] Kononen M, Hormia M, Kivilahti J, Hautaniemi J, Thesleff I (1992). Effect of surface processing on the attachment, orientation, and proliferation of human gingival fibroblasts on titanium. J Biomed Mater Res.

[35] Arima Y, Iwata H (2007). Effect of wettability and surface functional groups on protein adsorption and cell adhesion using well-defined mixed self-assembled monolayers. Biomaterials.

[36] Lai HC, Zhuang LF, Liu X, Wieland M, Zhang ZY, Zhang ZY (2010). The influence of surface energy on early adherent events of osteoblast on titanium substrates. J Biomed Mater Res A.

[37] Wang N, Li H, Wang J, Chen S, Ma Y, Zhang Z (2012). Study on the anticorrosion, biocompatibility, and osteoinductivity of tantalum decorated with tantalum oxide nanotube array films. ACS Appl Mater Interfaces.

[38] Hersel U, Dahmen C, Kessler H (2003). RGD modified polymers: biomaterials for stimulated cell adhesion and beyond. Biomaterials.

[39] Bartold PM, Narayanan AS (2006). Molecular and cell biology of healthy and diseased periodontal tissues. Periodontology.

[40] Anderson JM, Rodriguez A, Chang DT (2008). Foreign body reaction to biomaterials. Semin Immunol.

[41] Ku SH, Ryu J, Hong SK, Lee H, Park CB (2010). General functionalization route for cell adhesion on non-wetting surfaces. Biomaterials.

[42] Rozario T, DeSimone DW (2010). The extracellular matrix in development and morphogenesis: a dynamic view. Dev Biol.

[43] Valenick LV, Hsia HC, Schwarzbauer JE (2005). Fibronectin fragmentation promotes alpha4beta1 integrin-mediated contraction of a fibrin-fibronectin provisional matrix. Exp Cell Res.

[44] Veevers-Lowe J, Ball SG, Shuttleworth A, Kielty CM (2011). Mesenchymal stem cell migration is regulated by fibronectin through alpha5beta1-integrin-mediated activation of PDGFR-beta and potentiation of growth factor signals. J Cell Sci.

[45] Wang L, Zhou B, Liu Z, Dong L, Cheng K, Weng W (2018). Surface hydroxylation regulates cellular osteogeneses on TiO2 and Ta2O5 nanorod films. Colloids Surf B Biointerfaces.

[46] Chen W, Villa-Diaz LG, Sun Y, Weng S, Kim JK, Lam RH, Han L, Fan R, Krebsbach PH, Fu J (2012). Nanotopography influences adhesion, spreading, and self-renewal of human embryonic stem cells. ACS Nano.

[47] Abrahamsson I, Zitzmann NU, Berglundh T, Linder E, Wennerberg A, Lindhe J (2002). The mucosal attachment to titanium implants with different surface characteristics: an experimental study in dogs. J Clin Periodontol.

[48] Nevins M, Nevins ML, Camelo M, Boyesen JL, Kim DM (2008). Human histologic evidence of a connective tissue attachment to a dental implant. Int J Periodontics Restorative Dent.

[49] Nevins M, Kim DM, Jun SH, Guze K, Schupbach P, Nevins ML (2010). Histologic evidence of a connective tissue attachment to laser microgrooved abutments: a canine study. Int J Periodontics Restorative Dent.

[50] Nevins M, Camelo M, Nevins ML, Schupbach P, Kim DM (2012). Connective tissue attachment to laser-microgrooved abutments: a human histologic case report. Int J Periodontics Restorative Dent.

[51] Guillem-Marti J, Delgado L, Godoy-Gallardo M, Pegueroles M, Herrero M, Gil FJ (2013). Fibroblast adhesion and activation onto micro-machined titanium surfaces. Clin Oral Implants Res.

[52] den Braber ET, Jansen HV, de Boer MJ, Croes HJ, Elwenspoek M, Ginsel LA, Jansen JA (1998). Scanning electron microscopic, transmission electron microscopic, and confocal laser scanning microscopic observation of fibroblasts cultured on microgrooved surfaces of bulk titanium substrata. J Biomed Mater Res.

[53] Chou L, Firth JD, Uitto VJ, Brunette DM (1995). Substratum surface topography alters cell shape and regulates fibronectin mRNA level, mRNA stability, secretion and assembly in human fibroblasts. J Cell Sci.

[54] Khor HL, Kuan Y, Kukula H, Tamada K, Knoll W, Moeller M, Hutmacher DW (2007). Response of cells on surface-induced nanopatterns: fibroblasts and mesenchymal progenitor cells. Biomacromolecules.

